# Comprehensive Direct Georeferencing of Aerial Images for Unmanned Aerial Systems Applications

**DOI:** 10.3390/s22020604

**Published:** 2022-01-13

**Authors:** Carlos A. M. Correia, Fabio A. A. Andrade, Agnar Sivertsen, Ihannah Pinto Guedes, Milena Faria Pinto, Aline Gesualdi Manhães, Diego Barreto Haddad

**Affiliations:** 1 Federal Center of Technological Education of Rio de Janeiro (CEFET/RJ), Rio de Janeiro 20271-110, Brazil; carlos.correia@aluno.cefet-rj.br (C.A.M.C.); ihannah.guedes@aluno.cefet-rj.br (I.P.G.); milena.pinto@cefet-rj.br (M.F.P.); aline.manhaes@cefet-rj.br (A.G.M.); diego.haddad@cefet-rj.br (D.B.H.); 2 NORCE Norwegian Research Centre, 5838 Bergen, Norway; agsi@norceresearch.no; 3 Department of Microsystems, Faculty of Technology, Natural Sciences and Maritime Sciences, University of South-Eastern Norway (USN), 3184 Borre, Norway

**Keywords:** direct georeferencing, UAS, pinhole camera model

## Abstract

Optical image sensors are the most common remote sensing data acquisition devices present in Unmanned Aerial Systems (UAS). In this context, assigning a location in a geographic frame of reference to the acquired image is a necessary task in the majority of the applications. This process is denominated direct georeferencing when ground control points are not used. Despite it applies simple mathematical fundamentals, the complete direct georeferencing process involves much information, such as camera sensor characteristics, mounting measurements, attitude and position of the UAS, among others. In addition, there are many rotations and translations between the different reference frames, among many other details, which makes the whole process a considerable complex operation. Another problem is that manufacturers and software tools may use different reference frames posing additional difficulty when implementing the direct georeferencing. As this information is spread among many sources, researchers may face difficulties on having a complete vision of the method. In fact, there is absolutely no paper in the literature that explain this process in a comprehensive way. In order to supply this implicit demand, this paper presents a comprehensive method for direct georeferencing of aerial images acquired by cameras mounted on UAS, where all required information, mathematical operations and implementation steps are explained in detail. Finally, in order to show the practical use of the method and to prove its accuracy, both simulated and real flights were performed, where objects of the acquired images were georeferenced.

## 1. Introduction

Currently, optical image sensors are ubiquitous in Unammned Aerial Systems (UAS). These acquisition devices can be RGB, infrared or hyperspectral cameras, for example. There are a variety of applications using such sensors, e.g., in the fields of inspection [1], mapping [2], search and rescue [3], tracking [4], border patrol [5], sea surveillance [6], agriculture [7], and even recreation [8].

Traditionally, the georeferencing of aerial images was performed with the use of Ground Control Points (GCP) [9,10], which are marked points on the ground that have a known geographic location. However, the need of GCP imposes a considerable limitation in such operations because most of the time the operator cannot place and measure the GCP prior to the mission. For example, in [11], the authors developed a large-scale video fast geo-registration method for forest fire monitoring, using high-resolution camera mounted in UAS. The developed system uses selected terrain points as references, called “landmarks ground control points”, LGCPs, whose positions and geographic coordinates are known from official geographic maps and loaded in the system, in order to precede the obtained image georeferencing. Despite the method may be satisfactory for land monitoring, it is limited for terrains where the mapping was not accurate or known, as well in case there will be no possibility to chose LGCP, as deserts or maritime regions, for example.

The process of georeferencing aerial images without the use of GCPs is called Direct Georeferencing. This process relies on using information about the position, attitude, image sensor and lens characteristics to calculate the position of the aerial image object in the geographic frame. This involves many challenges, such as that there is no range information available, i.e., direct distance between the camera and the target.

One may say that this could be simply solved by equipping the UAS with laser scanners or stereo cameras. However, these sensors are costly for long ranges and would also increase the weight of the UAS payload, reducing its endurance.

In the literature, there are some works that perform direct georeferencing. Hemerly [12] has the merit of being one of the main references in the field, bringing a brief explanation of the direct georeferencing process, which is tested in a real UAS flight; the paper also mentions the camera calibration process. Leira et al. [13] implements a light-weight thermal camera for small fixed-wing UAS, with a georeferencing algorithm. The georeferencing process is explained and a real flight test is performed, where eighty images are georeferenced.

In [14], the authors proposed a real time direct georeferencing system for maritime targets, employing a thermal camera mounted on an UAS. The system is tested for a static target and for a moving vessel. All the steps of the georeferencing process are discussed. The algorithm is later used in [4] to calculate the target’s position for further tracking in two of the four tracking solutions presented with the use of UAS. Real flight tests were taken to evaluate the solutions. In [15], a rapid aerial image georeferencing method for UAS for agricultural activities was implemented. Low cost Inertial Measurement Unit (IMU) sensors, Global Positioning System (GPS) and sensor fusion techniques were employed, and GCPs were not used. The method obtains the georeferenced position by treating navigation data and the camera lens distortion model. Results were compared with sixteen positions given by GCP and the accuracy was considered satisfactory for agriculture applications.

In [16], the authors implement a method for estimate the coordinates of a target from the image obtained by a camera, mounted in a small fixed-wing UAS, given the UAS position and altitude, and the camera posed angles. The georeferencing calculations are similar to those presented in our research, which are detailed and fully explained here. The paper also brings four techniques to reduce the estimated georeferenced position error.

As observed in the previously cited works, although the method of direct georeferencing of images taken by cameras mounted on UAS demands simple mathematical concepts, all of them in linear algebra theory, the entire process involves many other details, resulting in a complex and tricky operation. Therefore, a researcher may face it difficult to find and comprehend these details, as they are treated separately and pulverized in many works in the scientific literature, increasing the risk of making small mistakes, which lead to big errors in the results. In fact, there is absolutely no paper in the literature that explain the process in a comprehensive way.

Therefore, this paper adds to the field by detailing the complete implementation of the direct georeferencing process, clarifying the concepts, and the reasons behind the choices. The method is tested both in real and simulated environments, as there has been significant growth on the use of simulation tools for UAS applications. In addition, the determination of the $z_{C}$ factor, briefly, or imprecisely, mentioned or not mentioned at all by the other works, is completely elucidated.

Another contribution of this paper is the inclusion of platform mounting measurements (lever arms) in the calculations. This is often neglected in the UAS community. Since the lever arms are in the range of centimeters, they are usually not considered. However, this can result on significant errors for high resolution sensors, or larger UAS.

Finally, the challenge that this work tries to overcome is the lack of centralized and standardized information about the methods to perform the georeferencing of aerial images taken by cameras mounted on UAS. It is important to well understand the methods because there are many different frames involved in the process and the tools provided by sensors and systems manufacturers may use different frames and axis orientations. Therefore, it is important to understand the mathematical fundamentals and the implementation steps to be able to consciously make the required conversions and adaptations.

## 2. Materials and Methods

In this section, the definition of direct georeferencing, as well as its applications will be presented. In addition, the most important mathematical tools used in the georeferencing, such as rotations and aircraft maneuver angles, will be formulated, in order to guarantee a self-contained material. Finally, the implementation steps will be described.

### 2.1. Direct Georeferencing

Georeferencing is the process of assigning to an object or target a location in a geographic frame of Reference [17]. In this paper, the objects of interest, or the targets, are initially available in an aerial image acquired by a monocular camera mounted on an UAS. The goal is, therefore, to calculate the location of the object in a geographic frame, such as the Universal Transverse Mercator frame or World Geodetic System 1984 frame. To perform these calculations, information about the camera sensor, mounting characteristics, and attitude and position of the UAS in the geographic frame is used, among other information. Considering that this process does not involve Ground Control Points, it is defined as Direct Georeferencing.

### 2.2. Mathematical Fundamentals

In this section, the mathematical support used in the direct georeferencing equations are presented. These comprises of Linear Algebra concepts, such as rotation matrices, and orientation angles fundamentals.

#### 2.2.1. Rotation Matrix

**Figure 1:**
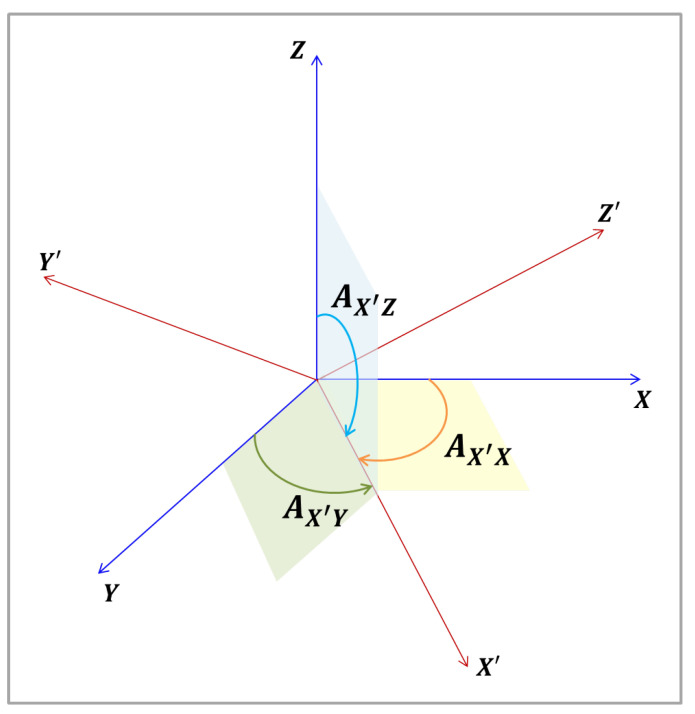
Angles between $X^{\prime }Y^{\prime }Z^{\prime }$ and XYZ axes.

Figure 1 shows two different coordinates systems, represented by the orthogonal reference frames XYZ and $X^{\prime }Y^{\prime }Z^{\prime }$. It also presents the angles between axis $X^{\prime }$ and each axis of the XYZ frame, which will be used in the coordinates transformations between the frames. The others angles that each axis forms with the axes of the other frame are not shown in Figure 1 for the didactic purposes.

From Figure 1, it can be seen that the unit basis vectors of $X^{\prime }$$Y^{\prime }$ and $Z^{\prime }$ axes ($\hat{i^{\prime }}$, $\hat{j^{\prime }}$ and $\hat{k^{\prime }}$) projections in the XYZ frame are given by the following Equations [18]:

(1) $$\hat{i^{\prime }}=\cos A_{X^{\prime }X}\hat{i}+\cos A_{X^{\prime }Y}\hat{j}+\cos A_{X^{\prime }Z}\hat{k},$$

(2) $$\hat{j^{\prime }}=\cos A_{Y^{\prime }X}\hat{i}+\cos A_{Y^{\prime }Y}\hat{j}+\cos A_{Y^{\prime }Z}\hat{k},$$

(3) $$\hat{k^{\prime }}=\cos A_{Z^{\prime }X}\hat{i}+\cos A_{Z^{\prime }Y}\hat{j}+\cos A_{Z^{\prime }Z}\hat{k},$$

where $A_{{\mathrm{axis}}^{\prime }-\mathrm{axis}}$ are the angles between the axis of the two frames, and ${\cos\;A}_{{\mathrm{axis}}^{\prime }-\mathrm{axis}}$ denotes the direction cosines of the vector on the axis.

These equations can be written in the matrix form as:

(4) $$\left[\begin{array}{c} \hat{i^{\prime }}\\ \hat{j^{\prime }}\\ \hat{k^{\prime }} \end{array}\right]=\left[\begin{array}{ccc} \cos A_{X^{\prime }X}\hat{i} & \cos A_{X^{\prime }Y}\hat{j} & \cos A_{X^{\prime }Z}\hat{k}\\ \cos A_{Y^{\prime }X}\hat{i} & \cos A_{Y^{\prime }Y}\hat{j} & \cos A_{Y^{\prime }Z}\hat{k}\\ \cos A_{Z^{\prime }X}\hat{i} & \cos A_{Z^{\prime }Y}\hat{j} & \cos A_{Z^{\prime }Z}\hat{k} \end{array}\right]=R_{XYZ\to X^{\prime }Y^{\prime }Z^{\prime }}\left[\begin{array}{c} \hat{i}\\ \hat{j}\\ \hat{k} \end{array}\right],$$

where the matrix $R_{XYZ\to X^{\prime }Y^{\prime }Z^{\prime }}$ is known as the Directional Cosine Matrix (DCM), or the rotation matrix, from the XYZ frame to the $X^{\prime }Y^{\prime }Z^{\prime }$ frame, which is given by:    

(5) $$R_{XYZ\to X^{\prime }Y^{\prime }Z^{\prime }}=\left[\begin{array}{ccc} \cos A_{X^{\prime }X} & \cos A_{X^{\prime }Y} & \cos A_{X^{\prime }Z}\\ \cos A_{Y^{\prime }X} & \cos A_{Y^{\prime }Y} & \cos A_{Y^{\prime }Z}\\ \cos A_{Z^{\prime }X} & \cos A_{Z^{\prime }Y} & \cos A_{Z^{\prime }Z} \end{array}\right].$$

Now, let *V* be a vector in the frame XYZ:

(6) $$V=a\hat{i}+b\hat{j}+c\hat{k}=\left[a\;b\;c\right]\left[\begin{array}{c} \hat{i}\\ \hat{j}\\ \hat{k} \end{array}\right].$$

Therefore, the vector $V^{\prime }$, which is the representation of the vector *V* on the frame $X^{\prime }Y^{\prime }Z^{\prime }$ can be obtained by:

(7) $$V^{\prime }=\left[\begin{array}{c} a^{\prime }\hat{i}\\ b^{\prime }\hat{j}\\ c^{\prime }\hat{k} \end{array}\right]=R_{XYZ\to X^{\prime }Y^{\prime }Z^{\prime }}V=\left[\begin{array}{ccc} \cos A_{X^{\prime }X} & \cos A_{X^{\prime }Y} & \cos A_{X^{\prime }Z}\\ \cos A_{Y^{\prime }X} & \cos A_{Y^{\prime }Y} & \cos A_{Y^{\prime }Z}\\ \cos A_{Z^{\prime }X} & \cos A_{Z^{\prime }Y} & \cos A_{Z^{\prime }Z} \end{array}\right]\left[\begin{array}{c} a\hat{i}\\ b\hat{j}\\ c\hat{k} \end{array}\right].$$

or:

(8) $$V^{\prime }=R_{XYZ\to X^{\prime }Y^{\prime }Z^{\prime }}V.$$

To perform the inverse transformation, that is, from the $X^{\prime }Y^{\prime }Z^{\prime }$ frame to XYZ frame, the rotation matrix $R_{X^{\prime }Y^{\prime }Z^{\prime }\to XYZ}$ is given by:

(9) $$R_{X^{\prime }Y^{\prime }Z^{\prime }\to XYZ}=\left[\begin{array}{ccc} \cos A_{XX^{\prime }} & \cos A_{XY^{\prime }} & \cos A_{XZ^{\prime }}\\ \cos A_{YX^{\prime }} & \cos A_{YY^{\prime }} & \cos A_{YZ^{\prime }}\\ \cos A_{ZX^{\prime }} & \cos A_{ZY^{\prime }} & \cos A_{ZZ^{\prime }} \end{array}\right].$$

Considering the congruence of the angles between axis of the two frames, given by:

(10) $$A_{X^{\prime }X}=A_{XX^{\prime }};\;A_{X^{\prime }Y}=A_{YX^{\prime }}\;;A_{X^{\prime }Z}=A_{ZX^{\prime }},$$

(11) $$A_{Y^{\prime }X}=A_{XY^{\prime }};\;A_{Y^{\prime }Y}=A_{YY^{\prime }}\;;A_{Y^{\prime }Z}=A_{ZY^{\prime }},$$

(12) $$A_{Z^{\prime }X}=A_{XZ^{\prime }};\;A_{Z^{\prime }Y}=A_{YZ^{\prime }}\;;A_{Z^{\prime }Z}=A_{ZZ^{\prime }},$$

one may conclude that:

(13) $$\begin{array}{ccc} R_{X^{\prime }Y^{\prime }Z^{\prime }\to XYZ} & = & \left[\begin{array}{ccc} \cos A_{XX^{\prime }} & \cos A_{XY^{\prime }} & \cos A_{XZ^{\prime }}\\ \cos A_{YX^{\prime }} & \cos A_{YY^{\prime }} & \cos A_{YZ^{\prime }}\\ \cos A_{ZX^{\prime }} & \cos A_{ZY^{\prime }} & \cos A_{ZZ^{\prime }} \end{array}\right] \end{array}$$

(14) $$\begin{array}{ccc} & = & \left[\begin{array}{ccc} \cos A_{X^{\prime }X} & \cos A_{Y^{\prime }X} & \cos A_{Z^{\prime }X}\\ \cos A_{X^{\prime }Y} & \cos A_{Y^{\prime }Y} & \cos A_{Z^{\prime }Y}\\ \cos A_{X^{\prime }Z} & \cos A_{Y^{\prime }Z} & \cos A_{Z^{\prime }Z} \end{array}\right]=R_{XYZ\to X^{\prime }Y^{\prime }Z^{\prime }}^{⊺}, \end{array}$$

or:

(15) $$R_{X^{\prime }Y^{\prime }Z^{\prime }\to XYZ}=R_{XYZ\to X^{\prime }Y^{\prime }Z^{\prime }}^{⊺}.$$

So, given the rotation matrix from one frame, said XYZ, to another, said $X^{\prime }Y^{\prime }Z^{\prime }$, in order to perform the inverse transformation, the new rotation matrix will be given by its transpose matrix:

(16) $$V^{\prime }=R_{XYZ\to X^{\prime }Y^{\prime }Z^{\prime }}V$$

(17) $$V=R_{X^{\prime }Y^{\prime }Z^{\prime }\to XYZ}V^{\prime }=R_{XYZ\to X^{\prime }Y^{\prime }Z^{\prime }}^{⊺}V^{\prime }.$$

It can also be demonstrated that, in the specific case of rotation matrices, the following statement is true:

(18) $$R_{XYZ\to X^{\prime }Y^{\prime }Z^{\prime }}^{⊺}R_{XYZ\to X^{\prime }Y^{\prime }Z^{\prime }}=R_{X^{\prime }Y^{\prime }Z^{\prime }\to XYZ}^{⊺}R_{X^{\prime }Y^{\prime }Z^{\prime }\to XYZ}=\left[\begin{array}{ccc} 1 & 0 & 0\\ 0 & 1 & 0\\ 0 & 0 & 1 \end{array}\right]=I,$$

where *I* canonically represents the identify matrix.

#### 2.2.2. Euler Angles Sequence for Aerodynamics: Yaw, Pitch and Roll

There is a mathematical method to transform coordinates from one frame to another, that is, to perform the rotations of vectors between two frames. This technique is known as Euler angles sequence, and consists to choose a sequence of three angles to project one frame into another. There are many possibilities for these sequences. In aerodynamics, a specific angles sequence is more convenient to use, because its relationship with the aircraft movements: yaw, pitch and roll.

**Figure 2:**
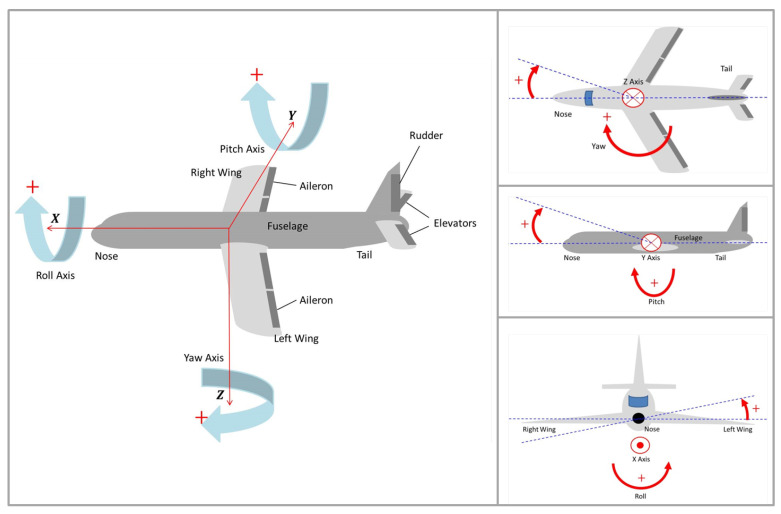
Airplane yaw, pitch and roll maneuvers.

The yaw movement is the turn to the left or right of the nose of the airplane, provoked by the action of the rudder. In other words, it is a rotation in the axis normal to the plane formed by the wings and the fuselage. The pitch movement consists of turning the nose up or down, and it is generated by the movement of the elevators, located in the tail of the airplane. This is equivalent to a rotation in an axis along the wings. Finally, the roll movement is the rotation along the airplane longitudinal axis, which is the axis along the fuselage. This maneuver is provided by the ailerons, placed at the rear of the wings [19]. Figure 2 shows the yaw, pitch and roll airplane maneuvers, with their respective axes.

As seen in Figure 2, the axes are canonically attributed, respectively, to *Z* axis, for the yaw, *Y* axis for the pitch and *X* axis for the roll movements. Its positive rotations are given by the right hand law, with the thumb aligned with the arrow of the axis.

**Figure 3:**
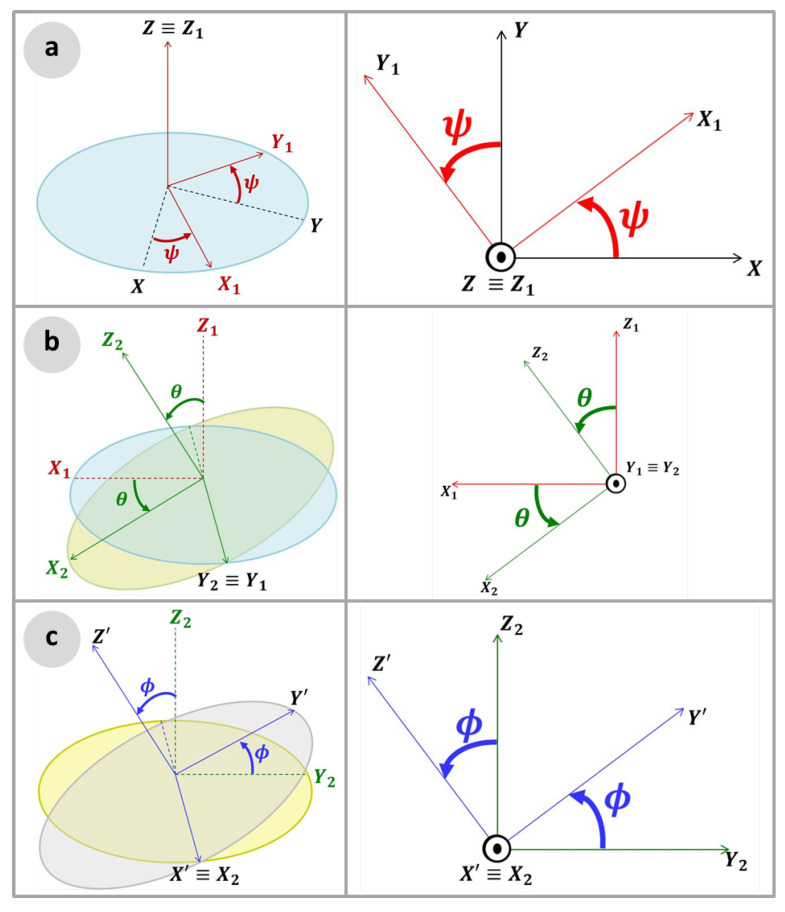
Euler sequence: (**a**) Yaw; (**b**) Pitch; and (**c**) Roll.

Figure 3 shows the Euler angles sequence for aerodynamics [18]. Figure 3a shows the first rotation around the *Z* axis, by the yaw angle ψ, which carries the frame XYZ to the frame $X_{1}Y_{1}Z_{1}$, where the axis *Z* is congruent to $Z_{1}$; Figure 3b presents the second rotation, around the *Y* axis, by the pitch angle θ, moving the frame $X_{1}Y_{1}Z_{1}$ to the frame $X_{2}Y_{2}Z_{2}$, where the axis $Y_{1}$ is congruent to $Y_{2}$; Figure 3c shows the third and last rotation, in the *X* axis, by the roll angle ϕ, rotating from the $X_{2}Y_{2}Z_{2}$ reference frame to the final $X^{\prime }Y^{\prime }Z^{\prime }$ frame. The notations ψ, θ and ϕ for the Euler angles yaw, pitch and roll, respectively, are canonical in the literature and, therefore, are employed in this work.

The rotation matrix for the Euler sequence yaw, pitch and roll angles, $R_{YPR}$, is given by [18]:

(19) $$R_{YPR}=\left[\begin{array}{ccc} \cos\psi \;\cos\theta & \sin\psi \;\cos\theta & -\sin\theta \\ \cos\psi \;\sin\theta \;\sin\phi -\;\sin\psi \;\cos\theta & \sin\psi \;\sin\theta \;\sin\phi +\cos\psi \;\cos\phi & \cos\theta \;\sin\phi \\ \cos\psi \;\sin\theta \;\cos\phi +\;\sin\psi \;\sin\phi & \sin\psi \;\sin\theta \;\cos\phi -\cos\psi \;\sin\phi & \cos\theta \cos\phi \end{array}\right].$$

The Euler angles values are given by:

(20) $$\tan\psi =\frac{\cos A_{XY^{\prime }}}{\cos A_{XX^{\prime }}}$$

(21) $$\sin\theta =-\cos A_{XZ^{\prime }}$$

(22) $$\tan\phi =-\;\frac{\cos A_{YZ^{\prime }}}{\cos A_{ZZ^{\prime }}}$$

### 2.3. Implementation

This section presents each step of the direct georeferencing process, which basically consists of successive rotations between the reference systems, or frames, involved in the chain.

#### 2.3.1. Image Frame to Camera Frame

**Figure 4:**
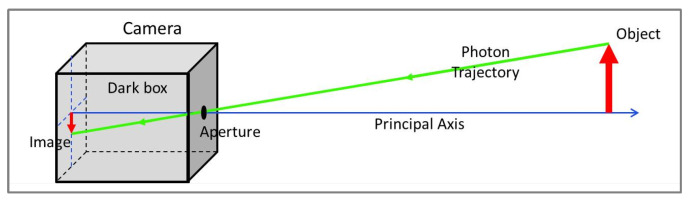
Camera Model.

Photographic cameras are devices that are able to record images from the three-dimensional world in bidimensional surfaces, or the image planes. Their construction is very simple, consisting of a dark box with a small aperture; the photons, reflected on the object surface in the three-dimensional world, will pass through the aperture and will form the image on the opposite surface of the dark box, where an optical sensor may be placed. Figure 4 shows this scheme.

**Figure 5:**
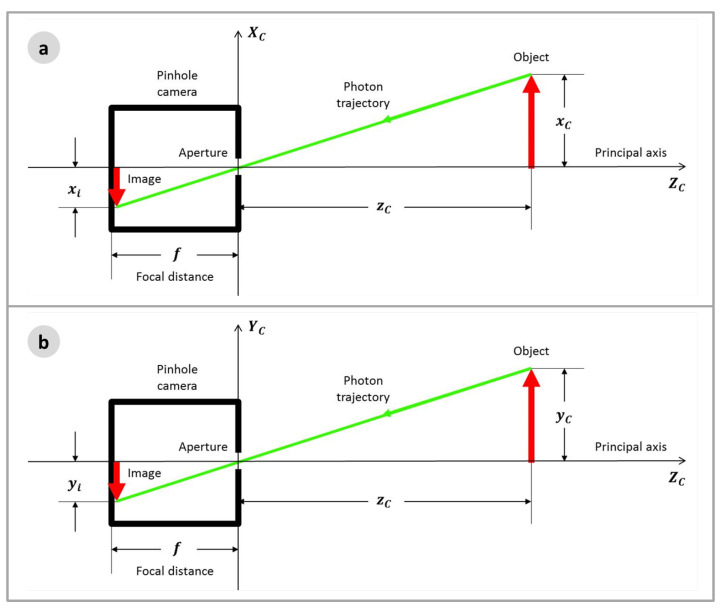
Pinhole Camera Model: (**a**) *X* axis view; and (**b**) *Y* axis view.

The system can be mathematically modelled by introducing a coordinate reference in the camera aperture, called Camera Frame (*C*), where the object position is plotted in coordinates $x_{C}$, $y_{C}$, $z_{C}$, and another reference frame, the Image Frame (*i*), placed on the image plane, or focal plane, where the image position is plotted in the coordinates ($x_{i}$, $y_{i}$). Figure 5 shows the model.

**Figure 6:**
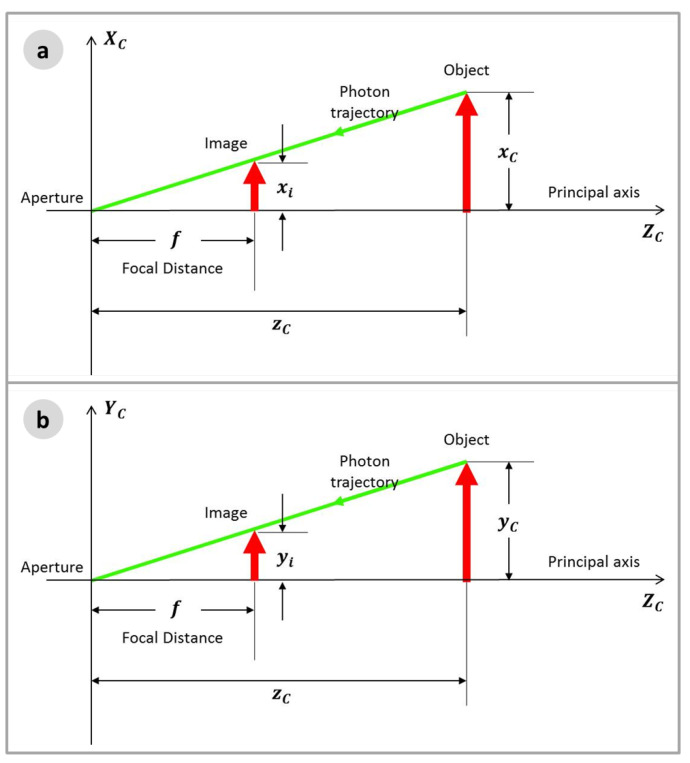
Equivalent Camera Model: (**a**) *X* axis view; and (**b**) *Y* axis view.

This model is known as the Pinhole Camera Model. To make easier mathematical operations, a geometrically equivalent construction is obtained by mirroring the image plane, or the projection plane, to place it on the other side of the Camera Frame. This simple procedure allows to work only with positive values of the axes. Figure 6 demonstrates the scheme.

From Figure 6a the relation between the coordinates $x_{i}$, in the Image frame (*i*), and $x_{C}$, in the Camera frame (*C*), is obtained, where *f* is the focal distance.

(23) $$\frac{x_{i}}{f}=\frac{x_{C}}{z_{C}}∴x_{i}=f\frac{x_{C}}{z_{C}}.$$

Furthermore, from Figure 6b:

(24) $$\frac{y_{i}}{f}=\frac{y_{C}}{z_{C}}∴y_{i}=f\frac{y_{C}}{z_{C}}.$$

It is important to note that these equations are only valid for rectified images. If the image is not rectified, a rectifying process is needed prior to the use of these equations.

**Figure 7:**
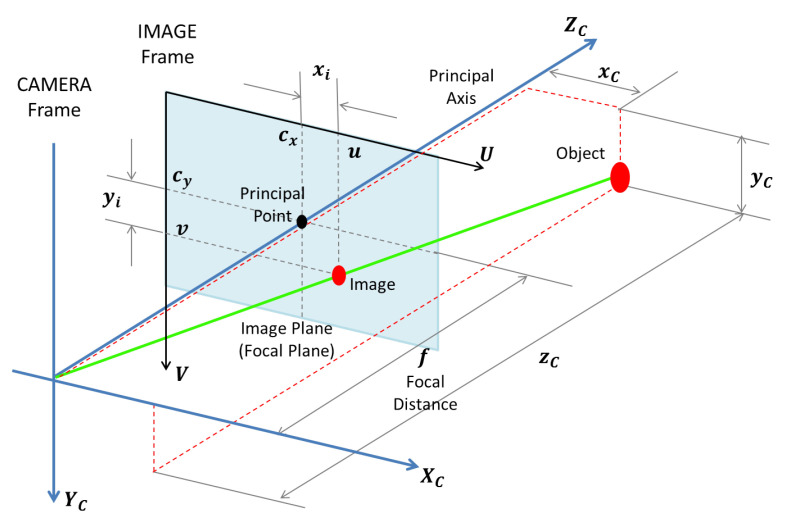
Equivalent Camera Model, in a more detailed perspective view, with the Image Frame top left orientation in the two axis *U* and *V*.

Figure 7 shows the same scheme in a more detailed perspective view. Here, the Image Frame is oriented in the two axis *U* and *V*:

(25) $$u-c_{x}=x_{i},$$

(26) $$vs.-c_{y}=y_{i},$$

where $c_{x}$ and $c_{y}$ are the coordinates of the principal point of the image.

Therefore, combining Equations (25) and (26) with Equations (23) and (24) leads to:

(27) $$u=f\frac{x_{C}}{z_{C}}+c_{x},$$

(28) $$vs.=f\frac{y_{C}}{z_{C}}+c_{y}.$$

In order to allow matrices operations, such as rotations and translations (the rigid transformations), which will be necessary in posteriors calculations, homogeneous coordinates will be employed. Therefore, Equations (27) and (28) take the following form:

(29) $$z_{C}\left[\begin{array}{c} u\\ v\\ 1 \end{array}\right]=\left[\begin{array}{ccc} f & 0 & c_{x}\\ 0 & f & c_{y}\\ 0 & 0 & 1 \end{array}\right]\left[\begin{array}{c} x_{C}\\ y_{C}\\ z_{C} \end{array}\right].$$

The coordinates *u* and *v* in the Image Frame *i* are conveniently used in pixels unit. The object coordinates in the Camera Frame (*C*) are usually given in meters. Therefore, naturally, the focal distance *f* and the coordinates of the principal point ($c_{x}$ and $c_{y}$) should be given in pixels.

The focal distance (*f*) is usually given in millimeters. Therefore, to convert to pixels, multiply by the Image Size in pixels and divide by the Sensor Size in millimeters:

(30) $$f\;\left[\mathrm{px}\right]=f\;\left[\mathrm{mm}\right]\frac{\mathrm{Image}\;\mathrm{Size}\;[\mathrm{px}]}{\mathrm{Sensor}\;\mathrm{Size}\;[\mathrm{mm}]}$$

In case of different pixel resolutions for *x* and for *y* dimensions, Equation (29) will have the form:

(31) $$z_{C}\left[\begin{array}{c} u\\ v\\ 1 \end{array}\right]=\left[\begin{array}{ccc} f_{x} & 0 & c_{x}\\ 0 & f_{y} & c_{y}\\ 0 & 0 & 1 \end{array}\right]\left[\begin{array}{c} x_{C}\\ y_{C}\\ z_{C} \end{array}\right].$$

Therefore, $f_{x}$ and $f_{y}$ in pixels should be calculated based on the focal length (*f* in millimeters) image dimensions (ImageWidth and ImageHeight in pixels) and sensor dimensions (SensorWidth and SensorHeight in millimeters):

(32) $$f_{x}=f\frac{\mathrm{ImageWidth}}{\mathrm{SensorWidth}},$$

(33) $$f_{y}=f\frac{\mathrm{ImageHeight}}{\mathrm{SensorHeight}}.$$

Equation (31) can also be written in the form:

(34) $$z_{C}P_{i}=KP_{C},$$

where $P_{i}={\left[u,v,1\right]}^{⊺}$ is the vector of the object’s coordinates in the Image Frame, $P_{C}={\left[x_{C},y_{C},z_{C}\right]}^{⊺}$ is the vector of the object’s coordinates in the Camera Frame, and *K* is the intrinsic parameters camera matrix, also referred as the calibration matrix, as it can be obtained during the camera calibration process.

(35) $$K=\left[\begin{array}{ccc} f_{x} & 0 & c_{x}\\ 0 & f_{y} & c_{y}\\ 0 & 0 & 1 \end{array}\right].$$

Equations (31) and (34) can be rewritten as:

(36) $$z_{C}\left[\begin{array}{c} u\\ v\\ 1 \end{array}\right]=K\left[\begin{array}{c} x_{C}\\ y_{C}\\ z_{C} \end{array}\right].$$

As the objective is to obtain the object’s metric coordinates in the camera frame from the object’s pixel coordinates in the image frame, Equation (36) then becomes:

(37) $$\left[\begin{array}{c} x_{C}\\ y_{C}\\ z_{C} \end{array}\right]=K^{-1}z_{C}\left[\begin{array}{c} u\\ v\\ 1 \end{array}\right].$$

Considering that $z_{C}$ is unknown, in order to resolve the equation, it will be incorporated to $P_{C}$, generating the $P_{C}^{\prime }$ vector:

(38) $$P_{C}^{\prime }=\left[\begin{array}{c} \frac{1}{z_{C}}x_{C}\\ \frac{1}{z_{C}}y_{C}\\ \frac{1}{z_{C}}z_{C} \end{array}\right]=\left[\begin{array}{c} x_{C}^{\prime }\\ y_{C}^{\prime }\\ 1 \end{array}\right]=K^{-1}\left[\begin{array}{c} u\\ v\\ 1 \end{array}\right].$$

As will be further demonstrated, $z_{C}$ will be easily determined at the end of the whole process, when the $P_{C}^{\prime }$ coordinates ($x_{C}^{\prime }$ and $y_{C}^{\prime }$), will be determined (as well the $P_{C}$ coordinates, $x_{C}$ and $y_{C}$). Until there, however, the process will follow using $P_{C}^{\prime }$.

#### 2.3.2. Camera Frame to Gimbal Frame

**Figure 8:**
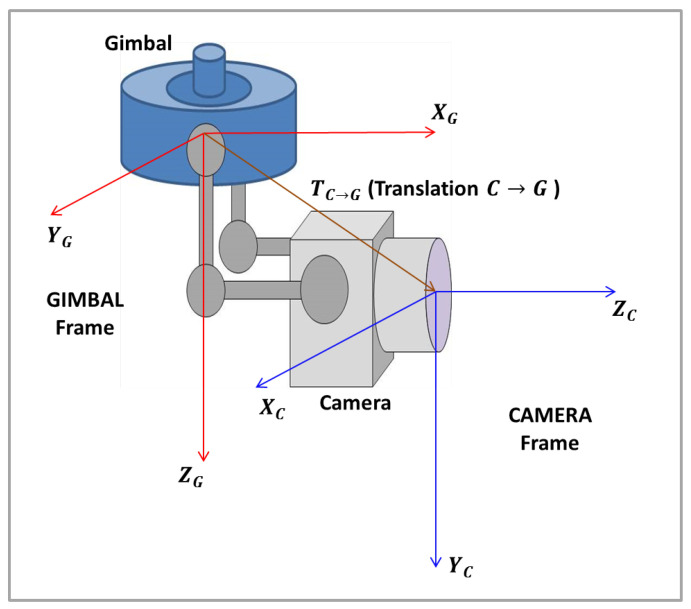
Camera and Gimbal frames.

The next step is the coordinates transformation from the Camera Frame (*C*) to the Gimbal Frame (*G*). The Gimbal Frame has its *X* axis, $X_{G}$, parallel to *Z* axis of the Camera Frame, $Z_{C}$; $Y_{G}$, the *Y* axis in Gimbal Frame, is parallel to the *X* axis of the Camera Frame, $X_{C}$; $Z_{G}$, the *Z* axis in the Gimbal Frame is parallel to the *Y* axis of the Camera Frame, $Y_{C}$. Figure 8 shows the Gimbal and the Camera frames.

The rotation matrix from the Camera Frame to the Gimbal Frame, $R_{C\to G}$, can be obtained throughout the projection of the Gimbal Frame in the Camera Frame, being given by:    

(39) $$R_{C\to G}=\left[\begin{array}{ccc} 0 & 0 & 1\\ 1 & 0 & 0\\ 0 & 1 & 0 \end{array}\right].$$

Therefore, the object’s position in the Gimbal Frame prime, $P_{G}^{\prime }$, is obtained by:

(40) $$P_{G}^{\prime }=R_{C\to G}P_{C}^{\prime },$$

or:

(41) $$\left[\begin{array}{c} x_{G}^{\prime }\\ y_{G}^{\prime }\\ z_{G}^{\prime } \end{array}\right]=\left[\begin{array}{ccc} 0 & 0 & 1\\ 1 & 0 & 0\\ 0 & 1 & 0 \end{array}\right]\left[\begin{array}{c} x_{C}^{\prime }\\ y_{C}^{\prime }\\ z_{C}^{\prime } \end{array}\right].$$

In cases where there is a difference in the position of the origin of the frames, as shown in Figure 8, a translation vector from the Camera Frame to the Gimbal Frame ($T_{C\to G}$) must be inserted. The object’s position in the Gimbal Frame prime will be then given by:

(42) $$P_{G}^{\prime }=R_{C\to G}P_{C}^{\prime }+T_{C\to G}.$$

#### 2.3.3. Gimbal Frame to UAS Frame

**Figure 9:**
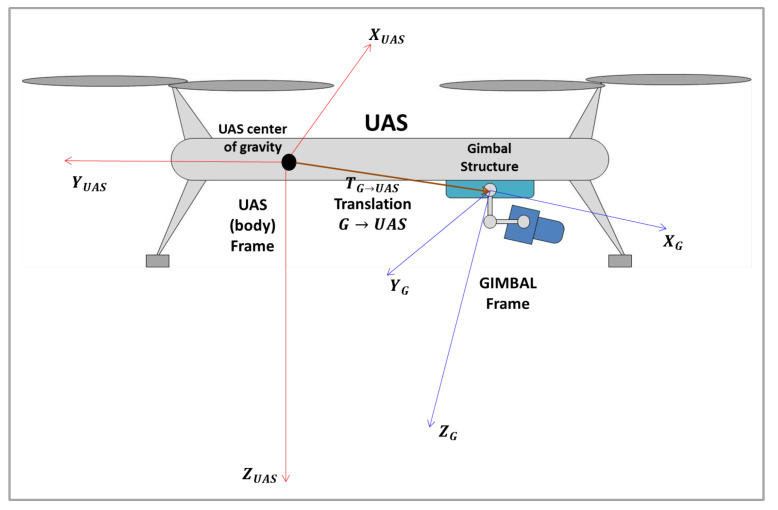
UAS and Gimbal frames.

The next step is the object’s coordinates transformation from Gimbal Frame to the UAS Frame, which is centered on the UAS center of gravity, as shown in Figure 9.

The transformation will require the Direction Cosine Matrix (DCM) from the Gimbal Frame to the UAS Frame, $R_{G\to UAS}$, given by the Euler angles yaw, pitch and roll. The operation is given by:

(43) $$P_{UAS}^{\prime }=\;R_{G\to UAS}P_{G}^{\prime }+T_{G\to UAS},$$

where $T_{G\to UAS}$ is the translation vector of the Gimbal Frame in the UAS Frame.

In a more complete form, Equation (43) may be written as:

(44) $$\begin{array}{ccc} P_{UAS}^{\prime } & = & R_{G\to UAS}P_{G}^{\prime }+T_{G\to UAS} \end{array}$$

(45) $$\begin{array}{ccc} & = & R_{G\to UAS}\left(R_{C\to G}P_{C}^{\prime }+T_{C\to G}\right)+T_{G\to UAS} \end{array}$$

(46) $$\begin{array}{ccc} & = & R_{G\to UAS}R_{C\to G}P_{C}^{\prime }+R_{G\to UAS}T_{C\to G}+T_{G\to UAS} \end{array}$$

#### 2.3.4. UAS Frame to NED Frame

**Figure 10:**
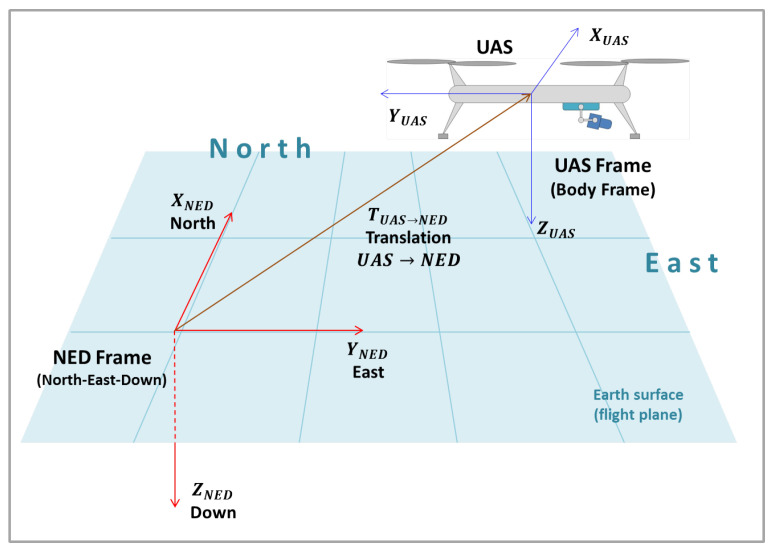
NED and UAS frames.

The following step is transform the object’s coordinates from the UAS Frame to the NED Frame (North-East-Down Frame). Figure 10 presents the scheme, also considering a translation vector $T_{UAS\to NED}$, which is the position of the origin of the UAS frame in the NED frame, if there is a difference between the origins of the reference systems.

To perform the coordinates transformation, the DCM between the frames, $R_{UAS\to NED}$, must be employed, and so, the Euler angles yaw, pitch and roll must be known. The pixel position in the NED frame prime, $P_{NED}^{\prime }$, is given by:

(47) $$P_{NED}^{\prime }=R_{UAS\to NED}P_{UAS}^{\prime }+T_{UAS\to NED}.$$

Substituting Equation (44) in Equation (47), in order to obtain a more complete form:

(48) $$\begin{array}{ccc} P_{NED}^{\prime } & = & R_{UAS\to NED}\left(R_{G\to UAS}R_{C\to G}P_{C}^{\prime }+R_{G\to UAS}T_{C\to G}+T_{G\to UAS}\right)+T_{UAS\to NED}\\ & = & R_{UAS\to NED}R_{G\to UAS}R_{C\to G}{P^{\prime }}_{C}+R_{UAS\to NED}R_{G\to UAS}T_{C\to G}\\ & & +R_{UAS\to NED}T_{G\to UAS}+T_{UAS\to NED} \end{array}$$

#### 2.3.5. NED Frame to ENU Frame

**Figure 11:**
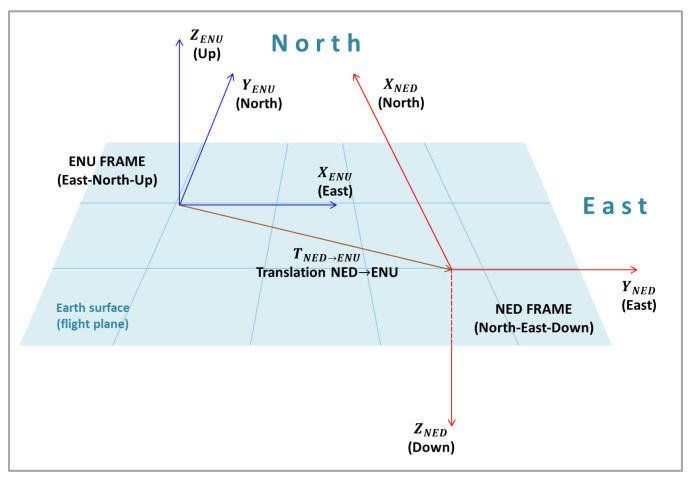
ENU and NED frames.

In many situations, it may be necessary to use the East-North-Up (ENU) reference system, as it is the reference frame employed in the Universal Transverse Mercator (UTM) coordinate system, which is directly associated with the World Geodetic System (WGS84) used by the Global Positioning Systems (GPS), with the well known latitude, longitude and height coordinates. Figure 11 presents the relation between the NED and the ENU frames.

From Figure 11, the rotation matrix from the NED frame to ENU frame, $R_{NED\to ENU}$, can be obtained:

(49) $$R_{NED\to ENU}=\left[\begin{array}{ccc} 0 & 1 & 0\\ 1 & 0 & 0\\ 0 & 0 & -1 \end{array}\right].$$

Thus, the primed position of the object in the ENU frame is given by:

(50) $$P_{ENU}^{\prime }=R_{NED\to ENU}P_{NED}^{\prime }+T_{NED\to ENU}.$$

Considering the previous calculations, a general equation may be extracted, substituting Equation (48) in Equation (50):

(51) $$\begin{array}{ccc} P_{ENU}^{\prime } & = & R_{NED\to ENU}R_{UAS\to NED}R_{G\to UAS}R_{C\to G}P_{C}^{\prime }\\ & & +R_{NED\to ENU}R_{UAS\to NED}R_{G\to UAS}T_{C\to G}\\ & & +R_{NED\to ENU}R_{UAS\to NED}T_{G\to UAS}\\ & & +R_{NED\to ENU}T_{UAS\to NED}+T_{NED\to ENU} \end{array}$$

To simplify, it can be written as:

(52) $$P_{ENU}^{\prime }=R_{C\to ENU}P_{C}^{\prime }+T_{C\to ENU},$$

where the matrix $R_{C\to ENU}$ is the product of all rotations:

(53) $$R_{C\to ENU}=R_{NED\to ENU}R_{UAS\to NED}R_{G\to UAS}R_{C\to G},$$

and vector $T_{C\to ENU}$ is the sum of all translations until this stage, considering the rotations in each step:

(54) $$\begin{array}{ccc} T_{C\to ENU} & = & R_{NED\to ENU}R_{UAS\to NED}R_{G\to UAS}T_{C\to G}\\ & & +R_{NED\to ENU}R_{UAS\to NED}T_{G\to UAS}\\ & & +R_{NED\to ENU}T_{UAS\to NED}\\ & & +T_{NED\to ENU}. \end{array}$$

#### 2.3.6. Determination of $z_{C}$

Equation (38) shows the relation between $P_{C}$ and $P_{C}^{\prime }$:

(55) $$P_{C}^{\prime }=\frac{1}{z_{C}}P_{C}.$$

Considering Equation (52), the object’s position in the ENU frame is given by:

(56) $$P_{ENU}=R_{C\to ENU}P_{C}+T_{C\to ENU}.$$

In order to determine $z_{C}$, it is necessary to solve the system formed by Equations (52) and (56):

(57) $$\left\{\begin{array}{c} P_{ENU}^{\prime }=R_{C\to ENU}P_{C}^{\prime }+T_{C\to ENU}\\ P_{ENU}=R_{C\to ENU}P_{C}+T_{C\to ENU} \end{array}\right.\;.$$

The second equation of the system in Equation (57) can be rewritten in the form:

(58) $$z_{C}P_{ENU}^{\prime }-z_{C}T_{C\to ENU}=R_{C\to ENU}P_{C}.$$

The combination of Equations (58) and (57) leads to:

(59) $$P_{ENU}=z_{C}P_{ENU}^{\prime }-z_{C}T_{C\to ENU}+T_{C\to ENU}.$$

**Figure 12:**
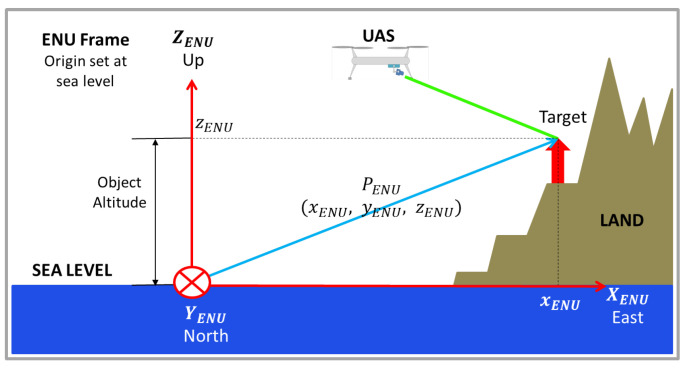
Object’s height in the ENU frame ($z_{ENU}$ coordinate).

Equation (59) is a matrix equation, and has solution in the *z* coordinates, because the $z_{ENU}$ coordinate, or the target object’s *z* coordinate in the ENU Frame, is known: it is the object’s altitude, considering that the origin of the ENU frame is set on sea level. In cases the ENU Frame is set on another level, the *z* coordinate will correspond to the vertical distance, as the height, from the origin of ENU frame to the object’s level. Figure 12 shows the scheme.

The value of $z_{ENU}$ must be known to perform the calculations, and it is very reasonable to assume this condition will be satisfied, because the region where the UAS is operating is known and so will be its altitude. In addition, digital elevation models can be used to estimate the altitude of the target.

Therefore, considering that $z_{ENU}^{\prime }$ and $z_{C\to ENU}$ had been determined in Equation (52), $z_{C}$ is finally given by:

(60) $$z_{C}=\frac{z_{ENU}-z_{C\to ENU}}{z_{ENU}^{\prime }-z_{C\to ENU}}.$$

Finally, the position of the object in the ENU frame can be obtained by using Equation (59)

(61) $$P_{ENU}=z_{C}P_{ENU}^{\prime }-z_{C}T_{C\to ENU}+T_{C\to ENU}$$

#### 2.3.7. Camera Matrix

Very often, in the literature, all the calculations demonstrated before are resumed in an unique Equation [4,12,13,14], in the form:

(62) $$z_{C}\left[\begin{array}{c} u\\ v\\ 1 \end{array}\right]=K\left[\begin{array}{cc} R_{ENU\to C} & T_{ENU\to C} \end{array}\right]\left[\begin{array}{c} x_{ENU}\\ y_{ENU}\\ \begin{array}{c} z_{ENU}\\ 1 \end{array} \end{array}\right]=M\left[\begin{array}{c} x_{ENU}\\ y_{ENU}\\ \begin{array}{c} z_{ENU}\\ 1 \end{array} \end{array}\right].$$

According to Hanning [20], Equation (62) is called pinhole model equation, where *M* is the camera matrix, used to denote a projective mapping from world coordinates ($x_{ENU}$, $y_{ENU}$, $z_{ENU}$) to pixel coordinates (u,v).

Seeking the goal of this work, which is to bring a complete and detailed exposition about the entire direct georeferencing process, a brief explanation about the meaning of this expression must be taken here.

The main aspect in this equation is the use of homogeneous coordinates, from the projective geometry, in place of the Euclidean geometry coordinates. This representation is necessary to allow calculations in matrix form, using a single matrix (the camera matrix *M*) when mapping a three-dimensional image from the real world to a bi-dimensional camera image, and vice versa [21].

In the equation, *K* is the intrinsic parameter matrix (35), $R_{ENU\to C}$ is the 3 × 3 matrix representing the product of all rotations from the Camera frame to the ENU frame, and $T_{ENU\to C}$ is the 3 × 1 matrix, which is the sum of all rotated translations from the ENU frame to the Camera frame.

According to Hanning [20], in homogeneous coordinates, the extrinsic matrix ($\left[\begin{array}{cc} R_{ENU\to C} & T_{ENU\to C} \end{array}\right]$) is given by:

(63) $$\left[\begin{array}{cc} R_{ENU\to C} & T_{ENU\to C} \end{array}\right]=\left[\begin{array}{cc} R_{ENU\to C} & T_{ENU\to C}\\ 0_{1\times 3} & 1 \end{array}\right]=\left[\begin{array}{ccc} r_{11} & r_{12} & \begin{array}{cc} r_{13} & t_{11} \end{array}\\ r_{21} & r_{22} & \begin{array}{cc} r_{23} & t_{21} \end{array}\\ \begin{array}{c} r_{31}\\ 0 \end{array} & \begin{array}{c} r_{32}\\ 0 \end{array} & \begin{array}{c} \begin{array}{cc} r_{33} & t_{31} \end{array}\\ \begin{array}{cc} 0 & \;\;\;1 \end{array} \end{array} \end{array}\right].$$

The camera matrix M is given by:

(64) $$M_{3\times 4}=\left[K\;0_{3\times 1}\right]\left[\begin{array}{cc} R_{ENU\to C} & T_{ENU\to C}\\ 0_{1\times 3} & 1 \end{array}\right]=\left[\begin{array}{ccc} f_{x} & 0 & \begin{array}{cc} c_{x} & 0 \end{array}\\ 0 & f_{y} & \begin{array}{cc} c_{y} & 0 \end{array}\\ 0 & 0 & \begin{array}{cc} 1 & \;\;0 \end{array} \end{array}\right]\left[\begin{array}{ccc} r_{11} & r_{12} & \begin{array}{cc} r_{13} & t_{11} \end{array}\\ r_{21} & r_{22} & \begin{array}{cc} r_{23} & t_{21} \end{array}\\ \begin{array}{c} r_{31}\\ 0 \end{array} & \begin{array}{c} r_{32}\\ 0 \end{array} & \begin{array}{c} \begin{array}{cc} r_{33} & t_{31} \end{array}\\ \begin{array}{cc} 0 & \;\;\;1 \end{array} \end{array} \end{array}\right].$$

As it can be seen, the intrinsic matrix *K* has also to be used in homogeneous coordinates. The completion of the matrices with zeros and ones is done in a way that the result of the calculations is the same before and after the homogeneous coordinates transformation.

### 2.4. Lens Camera Distortion

In this paper, the image used in the direct georeferencing process is assumed rectified. This means that no image distortion is present. Usually, manufacturers of UAS cameras provide the option to directly obtain the rectified image. However, as this subject may be an issue, it is briefly discussed in this section.

The geometry of the perspective or pinhole camera is simple since we assume the pinhole to be infinitely small. In reality, the light passes through a lens that complicates the camera model. The lens can distort the light rays projected onto the 2D sensor and this geometric effect of image distortion is important to compensate for with regard to georeferencing. A camera with radial distortion is not well described by the pinhole model. Many wide-angle lenses have noticeable radial distortion which basically means that lines in the scene appear as curves in the image. There are two types of radial distortion: (1) barrel distortion and (2) pincushion distortion. There are also other forms of distortion effects, such as tangential distortion.

By only considering barrel distortion, the distorted coordinate on the sensor plane can be represented as [22]:

(65) $$\begin{array}{cc} x_{distorted} & =x(1+k_{1}r^{2}+k_{2}r^{4}+k_{3}r^{6})\\ y_{distorted} & =y(1+k_{1}r^{2}+k_{2}r^{4}+k_{3}r^{6}) \end{array}$$

where $r=\sqrt{{\left(x-c_{x}\right)}^{2}+{\left(y-c_{y}\right)}^{2}}$, $c_{x}$ and $c_{y}$ is the optical centre pixel on the sensor and $k_{1}$, $k_{2}$ and $k_{3}$ are the radial distortion coefficients, which can be obtained during the camera calibration process. Therefore, the original raw distorted image can be rectified.

### 2.5. Software Implementation

In this section, a pseudocode to perform the direct georeferencing and the main functions used in the MATLAB and Python script are be presented. The structures followed in each script are the same and follow the six steps discussed in Section 2.3. For each implementation script, the specific employed functions will be explained, as well as its special procedures.

#### 2.5.1. Pseudocode

The steps discussed in the previous section can be summarized in Algorithm 1, that can be further implemented in any chosen programming language.
**Algorithm 1** Direct Georeferencing1:Input $P_{i}$2:Define *K*3:$P_{C}^{\prime }\leftarrow inverse\left(K\right)P_{i}$4:Define $R_{C\to G}$5:Input $T_{C\to G}$6:$P_{G}^{\prime }\leftarrow R_{C\to G}P_{C}^{\prime }+T_{C\to G}$7:Input $\psi_{G},\theta_{G},\phi_{G}$8:$R_{G\to UAS}\leftarrow transpose\left(DCM\left(\psi_{G},\theta_{G},\phi_{G},{}^{\prime \prime }ZYX^{\prime \prime }\right)\right)$9:Input $T_{G\to UAS}$10:$P_{UAS}^{\prime }\leftarrow R_{G\to UAS}P_{G}^{\prime }+T_{G\to UAS}$11:Input $\psi_{UAS},\theta_{UAS},\phi_{UAS}$12:$R_{G\to UAS}\leftarrow transpose\left(DCM\left(\psi_{UAS},\theta_{UAS},\phi_{UAS},{}^{\prime \prime }ZYX^{\prime \prime }\right)\right)$13:Input $T_{UAS\to NED}$14:$P_{NED}^{\prime }\leftarrow R_{UAS\to NED}P_{UAS}^{\prime }+T_{UAS\to NED}$15:Define $R_{NED\to ENU}$16:Input $T_{NED\to ENU}$17:$P_{ENU}^{\prime }\leftarrow R_{NED\to ENU}P_{NED}^{\prime }+T_{NED\to ENU}$18:$T_{C\to ENU}\leftarrow T_{NED\to ENU}+R_{NED\to ENU}T_{UAS\to NED}+R_{NED\to ENU}R_{UAS\to NED}T_{G\to UAS}+R_{NED\to ENU}R_{UAS\to NED}R_{G\to UAS}TC\to G$19:$z_{C\to ENU}\leftarrow T_{C\to ENU}\left[2\right]$20:$z_{ENU}^{\prime }\leftarrow P_{ENU}^{\prime }\left[2\right]$21:Input $z_{ENU}$22:$z_{C}=\left(z_{ENU}-z_{C\to ENU}\right)/\left(z_{ENU}^{\prime }-z_{C\to ENU}\right)$23:$P_{ENU}\leftarrow z_{C}P_{ENU}^{\prime }-z_{C}T_{C\to ENU}+T_{C\to ENU}$24:Output $P_{ENU}$

#### 2.5.2. MATLAB Implementation

The first step is the transformation from the Image to the Camera frame, and, in the beginning of all the calculations, the intrinsic parameters matrix data needs to be input. The intrinsic parameter matrix *K* is a 3-by-3 matrix, therefore in MATLAB has the following format: “K = [fx 0 cx; 0 fy cy; 0 0 1]”.

The pixel position coordinates in the Image Frame, *u* and *v*, are the next data required. The pixel position matrix in the Image Frame, $P_{i}$, is then determined. The MATLAB command “K\” gives the product of the inverse matrix of the intrinsic matrix K with the following term.

The following operations are matrix multiplication for rotations and sum for translations, which in MATLAB is done in a straightforward way.

In the transformation from the Gimbal to the UAS frame, in order to obtain the DCM for the rotation, the Euler angles sequence, yaw, pitch and roll must be input. The MATLAB function “angle2dcm(angl1, angl2, angl3)” gives the DCM matrix, where the parameters “angl1”, “angl2” and “angl3” are the aerodynamics Euler angles sequence, respectively, yaw, pitch and roll angles in this work. Here, it is important to note that the angles of the Euler sequence, yaw, pitch and roll, are commonly given in the most external reference frame, which in this case is the UAS Frame. Therefore, the MATLAB function will return the rotation from the UAS to the Gimbal frame. The rotation from the Gimbal to the UAS frame ($R_{G\to UAS}$) can be obtained by transposing the rotation matrix from the UAS to the Gimbal frame ($R_{UAS\to G}$), using the MATLAB function “transpose($R_{UAS\to G}$)”. This same procedure needs to be applied when obtaining the rotation from the UAS to the NED frame.

The last step of the script consists of finding the target’s position in the ENU frame. The coordinate $z_{ENU}$, which is equivalent to the distance from the target to the origin of the ENU frame in the *z* axis, needs to be input. It is also necessary to determine the total translation matrix, $T_{C\to ENU}$, which is given by the sum of all translations matrices, rotated with its related DCMs. Finally, to calculate $z_{C}$, $z_{ENU}^{\prime }$ and $z_{C\to ENU}$ are needed. In MATLAB, they are obtained by $P_{ENU}^{\prime }\left(3\right)$ and $T_{C\to ENU}\left(3\right)$, respectively.

#### 2.5.3. Python Implementation

In the Python implementation, NumPy [23] arrays are used for the matrices. As an example, to input a NumPy array for the intrinsic matrix, the following command is used: “K = numpy.array([[f_x, 0, c_x],[0, f_x, c_y],[0, 0, 1]])”.

With the NumPy library, it is easy to get the inverse matrix of the intrinsic matrix by using the “numpy.linalg.inv(K)” command.

The matrix multiplication is done by using the operator “@” that was introduced in Python 3.5, for example: “C = A @ B”.

The NavPy [24] library is used to provide the DCM matrices by the function “navpy. angle2dcm” which inputs are the three angles of rotation (yaw, pitch and roll). The default rotation sequence is the “*ZYX*” sequence, which is the one used by this work. In addition, the documentation says that the DCM transforms a vector from the locally level coordinate frame (i.e., the NED frame) to the body frame. Therefore, in this work the transpose of the given DCM will be used, which transforms the vector from the body frame to the NED frame.

Finally, in the last step, where $z_{C}$ is calculated, in order to access the third element of a NumPy array, the 2 index is used, for example: “z_T = T_T[2]”.

## 3. Results

To evaluate the proposed direct georeferencing method, two cases were explored. First, a target in picture taken in a simulation environment was georeferenced. Second, a real setup was used to take a picture of a target with a camera mounted on an UAS, which was then georeferenced.

### 3.1. Simulation Environment Example with UE4 and AirSim

**Figure 13:**
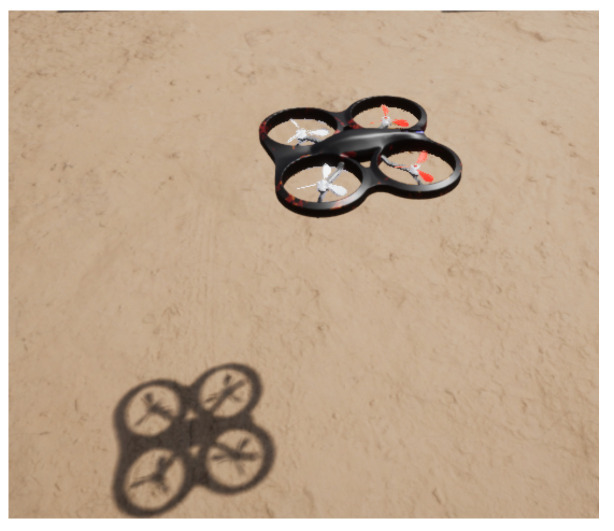
UAS in simulation environment.

In this case, a simulation environment was created in the Unreal Engine 4 (UE4). The AirSim plugin was used to simulate the UAS camera and flight dynamics. The UAS model in the simulation environment can be seen in Figure 13.

**Figure 14:**
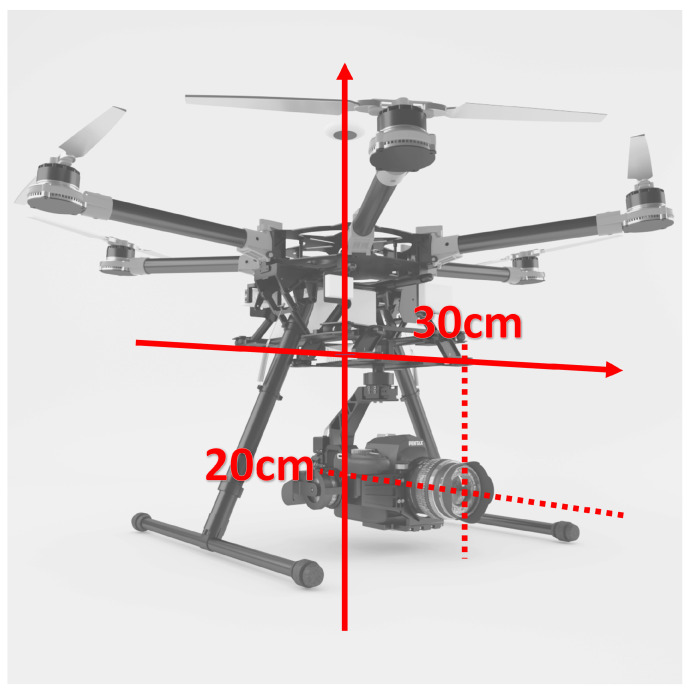
Camera placement on UAS in the simulation application.

The virtual camera was placed 20 cm down to the drone and 30 cm to the front as illustrated with the example of Figure 14.

**Figure 15:**
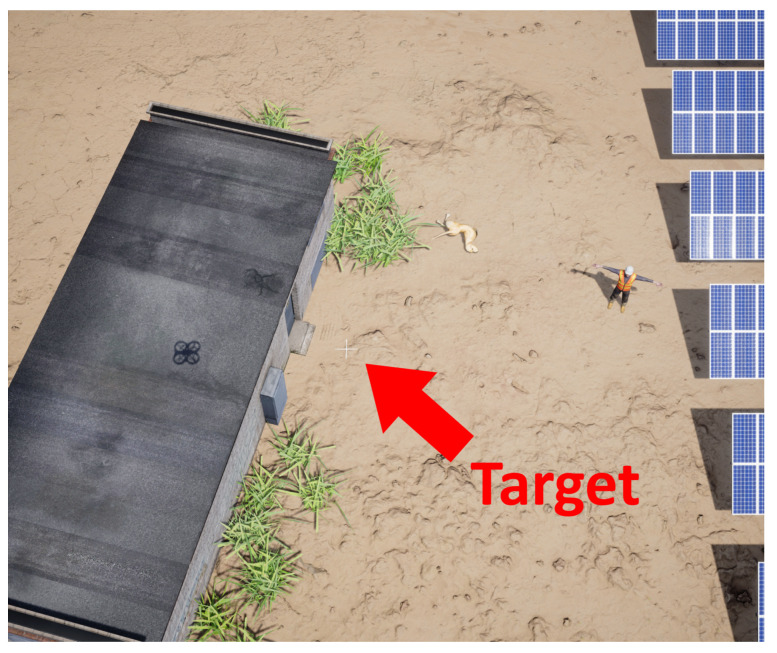
Picture acquired in the simulation environment.

A target (white cross) was placed on the ground, in front of the door of a power house (Figure 15). To simulate the Universal Transverse Mercator (UTM) frame, a random location in the simulation environment was chosen to be the origin of the UTM frame. The target’s position in virtual UTM coordinates was then 8.5 m to the east, −8.0 m to the north and 0 m of height, as it is positioned on the ground.

The gimbal’s attitude was configured to have a yaw ($\psi_{G}$) of −90 degrees (−π/2 radians) and a pitch ($\theta_{G}$) of −60 degrees (−π/3 radians) with respect to the world. The UAS was moved to a place where it could have a good view of the target and the surroundings to take the picture. The position of the drone ($T_{NED\to ENU}$ in meters) was given by the simulated sensors.

The camera was modeled based on the FLIR Chameleon 3 [25] camera sensor and a lens with 12.5 mm of focal distance. The camera has a resolution of 2448 × 2048 pixels and a sensor of 8.6 mm of width [25].

Additionally to the pixels resolution, the AirSim plugin models the camera by the horizontal Field of View in degrees. The following equation was used to calculate the horizontal field of view ($FOV_{\mathrm{horizontal}}$ in degrees) from the horizontal sensor size (SensorWidth in millimeters) and the focal length (*f* in millimeters):

(66) $$FOV_{\mathrm{horizontal}}=2\arctan\left(\frac{\mathrm{SensorWidth}}{2f}\right)$$

Therefore, for 8.6 mm of sensor width and 12.5 mm of focal length, the horizontal field of view is equal to 37.97 degrees.

The target (white cross) in Figure 15 is located on [u,v]=[1095,1099] pixels. The values of all parameters can be seen in Table 1.

The calculated georeferenced position of the target in the ENU frame ($P_{ENU}$) was [8.50283,−7.99841] m. This gives 100% of accuracy if rounded to the second decimal digit, proving that the georeferencing method is correct.

### 3.2. Practical Example with ROS

For the practical experiment, a ZED 2 camera [26] was mounted on the UAS pointing straight down without a gimbal. The Robot Operating System (ROS) was used to capture and save the data.

The camera ROS wrapper provides the topics of “image_rect_color”, where it is possible to obtain the rectified image; and the “camera_info”, where the camera intrinsic matrix *K* for the rectified image can be obtained.

The camera system is equipped with an IMU and a Magnetometer which are used in this work to calculate the UAS’ attitude. These sensors are mounted close to the camera sensor. The distance between the attitude sensors and the camera ($T_{G\to UAS}$) and the orientation between the camera and the attitude sensors ($\left[\psi_{G},\theta_{G},\phi_{G}\right]$) are given by the manufacturer in the ROS wrapper through the topic “left_cam_imu_transform”.

**Figure 16:**
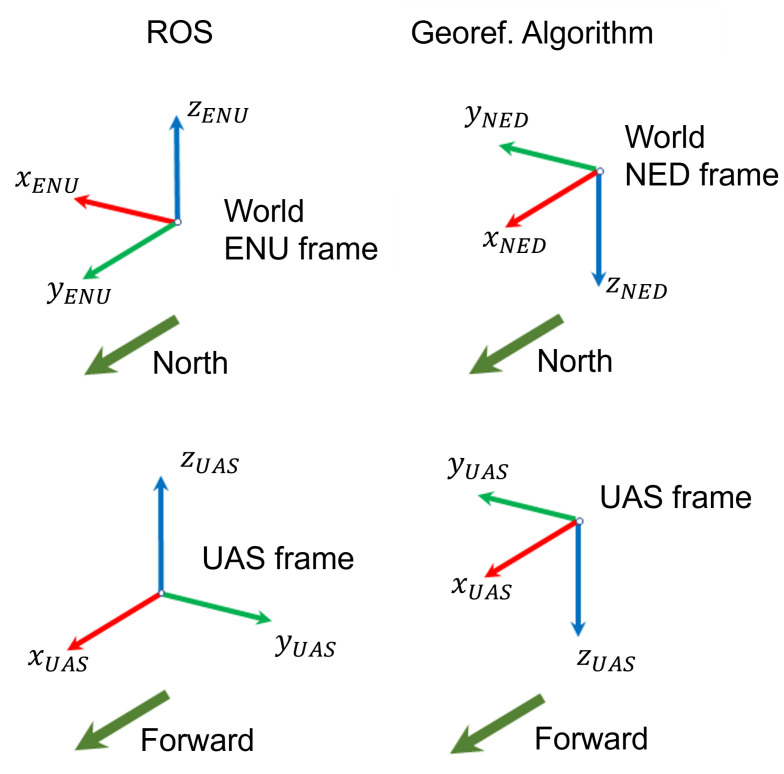
ROS and georeferencing algorithm frames.

The attitude is given in quaternions format in the topic “imu/data”. As Euler angles are used in this work, a conversion from quaternions to angles is needed. However, in order to proceed with the georeferencing calculations, additional conversions are required as the frame in ROS have different orientation than the UAS frame used by this work (Figure 16), which is the same used by flight control units. Therefore, as the *y* and *z* axes in ROS’ frame are pointing to the opposite direction of the axes *y* and *z* in this work’s UAS frame, the pitch angle obtained from the “imu/data” ROS topic needs to be inverted; and the yaw angle needs to be inverted and added 90 degrees (π/2 radians).

**Figure 17:**
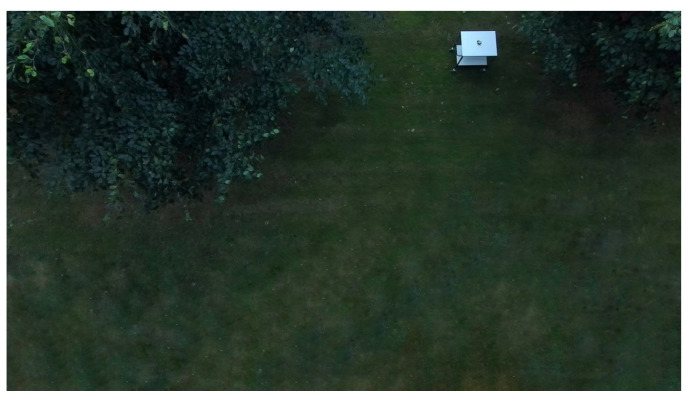
Aerial image taken by camera mounted on UAS.

The test setup was to take a picture (Figure 17) of the target object, which is a table of 80 × 60 × 85 cm.

The parameters considered for the direct georeferencing can be seen in Table 2. The translation from camera to gimbal frames ($T_{C\to G}$) is zero because there is no gimbal installed in this setup. To keep the same implementation steps that were described in the previous sections, as no gimbal was installed, the gimbal angles ($\left[\psi_{G},\theta_{G},\phi_{G}\right]$) considered in the calculations are actually the angles of the camera attitude with respect to the IMU (UAS frame). The same is valid for the translation ($T_{G\to UAS}$). The attitude of the IMU (UAS frame) is obtained from converting the quaternions from the ROS topic “imu/data” to Euler angles and making the required conversions from the frame used in ROS to the frame used in this work. The NED frame’s origin is considered at the same place as the IMU (UAS frame) and the ENU horizontal origin is placed on the UAS. Finally, the vertical ENU origin was placed on the ground, therefore, the height of the UAS ($z_{NED\to ENU}$) was obtained from its relative height (height from take-off).

**Figure 18:**
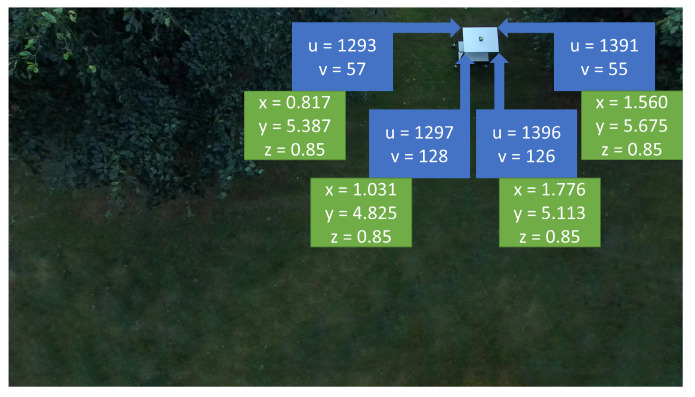
Pixel and georeferenced positions of table’s top shelf’s four corners.

The strategy used to evaluate the method was to georeference the four corners of the table’s top shelf. As it can be seen in Figure 18, the blue squares have the corners’ pixel coordinates and the green squares have the calculated georeferenced coordinates in the ENU frame. It is important to notice that in this case, as the corners of the top shelf are being georeferenced, the height of the object ($z_{ENU}$) is of 0.85 m.

The calculated lengths from corner to corner can be seen in Table 3. This was the strategy chosen to evaluate the algorithm as there was no way to precisely measure the position of the table during the experiments. Therefore, the georeferenced position presented in Figure 18 is relative to the UAS, as it can be observed in Table 2 from the $T_{NED\to ENU}$ translation vector. The result was very satisfactory as the calculated values perfectly match the dimensions of the shelf.

Finally, to evaluate the proposed solution’s performance when georeferencing targets at different heights, the distance between corners that are placed on top of each other was calculated and compared with the measured distance.

The bottom right corner of the top shelf is assumed at 0.85 m of height ($z_{ENU}$) and the bottom right corner of the bottom shelf is assumed at 0.35 m of height.

**Figure 19:**
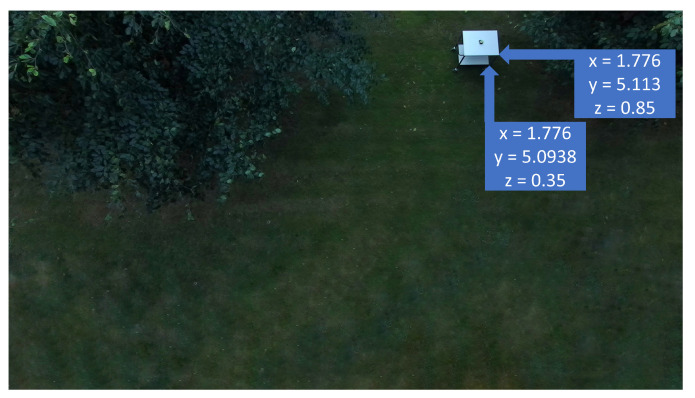
Georeferenced positions of bottom right corner of the top shelf and bottom right corner of the bottom shelf.

The georeferenced coordinates of both corners were calculated (Figure 19) and used to calculate the distance between them. The calculated distance was of 0.52 m, against 0.50 m of measured distance. Therefore, there was an error of 2 cm, which is small and could have happened because of the shelf’s thickness or deviations when manually choosing the pixels in the image.

## 4. Discussion

There are many different orientation frames in the process of direct georeferencing aerial images acquired by cameras mounted on UAS. First, the image frame, then the camera, gimbal, UAS (or IMU) and the geographic/world (NED and ENU) frames. In this work, in order to follow the literature and most common applications, two world frames were considered: the NED (North-East-Down); and ENU (East-North-Up) frames.

Despite camera, gimbal and UAS, NED and ENU frames were presented in this work, not all applications will require the use all these frames. Therefore, it is important to apply the method with the needed changes in each case.

As seen in the practical case study, it is important to understand the formats of the attitude data given by the flight control units or ROS, to be able to convert to the formats used in the direct georeferencing algorithm presented in this work. This is one of the reasons why this work is considered relevant as it explains the implementation steps so that the readers can understand the reasons behind the equations, in order to adapt the solution to their use.

In addition, it is important to note that accurate measurements are important for an accurate direct georeferencing. Therefore, GNSS RTK solutions should be used, in addition to highly accurate Inertial Measurement Units (IMU), magnetometers and gimbals. Furthermore, platform mounting measurements between the origins of the frames of reference (lever arms) should be included in the calculations as translation vectors. This is often neglected in the UAS community, since the lever arms are short, in the range of centimeters. However, for high resolution sensors in the order of millimeters, or larger UAS or manned aircraft, the lever arms are necessary to account for.

Another important aspect to consider is that depending on the IMU orientation, there may be problems when computing the yaw angles when the camera is pointing down. A possible solution for this issue is to change the orientation of the *z* axis so that it is perpendicular to the world when the UAS is horizontally positioned. A way to eliminate this issue is to use quaternions in all steps of the direct georeferencing implementation. This could be interesting future work.

## 5. Conclusions

This work presented a comprehensive method for direct georeferencing of aerial images taken by monocular cameras mounted on UAS. To perform the calculations, the method uses data about the camera sensor, relative position and orientation between camera, gimbal, UAS and world, and position and attitude sensor data. Two case studies were performed to show the method’s employment: georeferencing of a target in an image taken in a simulation environment; and georeferencing an object from an image taken in a real flight. For an image taken in a simulation environment, the georeferenced target position was precisely calculated. In the practical experiment, where the corners of a table were georeferenced, there were small inaccuracies, which were expected, as in real life setups there are small terrain deviations, sensor noise, among other problems. Therefore, the main goal of this work, which was to present a comprehensive direct georeferencing method and its implementation, was accomplished. This included the mathematical fundamentals and the assessment of practical challenges explored in the case studies. 

## Figures and Tables

**Table 1:** Parameters used for the direct georeferencing of the aerial imaged acquired in the simulation environment.

Parameter	Value	Note
$P_{i}$	[1095,1099]	
*K*	$\left[\begin{array}{ccc} 3558.1395 & 0 & 1224\\ 0 & 3558.1395 & 1024\\ 0 & 0 & 1 \end{array}\right]$	
** $T_{C\to G}$ **	[0,0,0]	The camera and the gimbal frames have the same origin.
$\left[\psi_{G},\theta_{g},\phi_{G}\right]$	[−π/2,−π/3,0]	
** $T_{G\to UAS}$ **	[0.3,0,0.2]	
$\left[\psi_{UAS},\theta_{UAS},\phi_{UAS}\right]$	[0,0,0]	As the gimbal’s attitude is with respect to the world, the UAS attitude is not considered.
** $T_{UAS\to NED}$ **	[0,0,0]	The UAS and NED frames have the same origin.
** $T_{NED\to ENU}$ **	[31.72212,−6.55099,42.44889]	
$z_{ENU}$	0	

**Table 2:** Parameters used for the direct georeferencing of the aerial imaged acquired in a real flight.

Parameter	Value	Note
*K*	$\left[\begin{array}{ccc} 1055.33422 & 0 & 1055.33423\\ 0 & 990.06824 & 544.24640\\ 0 & 0 & 1 \end{array}\right]$	
** $T_{C\to G}$ **	[0,0,0]	
$\left[\psi_{G},\theta_{G},\phi_{G}\right]$	[0.00176,0.00116,0.00138]	
** $T_{G\to UAS}$ **	[−0.002,0.023,0.002]	
$\left[\psi_{UAS},\theta_{UAS},\phi_{UAS}\right]$	[6.04630,−1.46643,−0.10614]	
** $T_{UAS\to NED}$ **	[0,0,0]	The UAS and NED frames have the same origin.
** $T_{NED\to ENU}$ **	[0,0,8.88]	

**Table 3:** Calculated length between shelf corners.

	Length [m]
top left to top right	0.80
top left to bottom left	0.60
top right to bottom right	0.60
bottom left to bottom right	0.80

## Data Availability

The MATLAB and Python codes, as well as the image data used in this work are openly available in https://github.com/professorfabioandrade/georef (accessed on 23 December 2021).
